# Spina Bifida Cured by Ligature

**Published:** 1837

**Authors:** William Judkins


### Art. II.—A Case of Spina Bifida, Cured by the Ligature. Communicated by William Judkins, M. D.

On the last day of 10th month, 1334, M. S. was delivered of a female child, affected with *Spina Bifida.* The tumor was about the size of a large hen's egg; situated over the lowest cervical vertebra, and evidently communicating with the cavity of the spinal theca. In all other respects the child was perfect and healthy. I advised the parents to let the tumor alone, and if the child should live for a few months, I would endeavor to do something for its relief. It grew finely, and had a regular and healthful developement of all its organs.—But the tumor grew in a corresponding ratio, and at the end of three months, I called in Dr. Drake, who examined it carefully, and on finding that it could be nearly emptied by pressure, and that the child manifested great uneasiness when the contents of the sack were forced in the direction of the spinal cord, did not hesitate to concur in the opinion I had formed of it; and at the same time advised that I should still omit any application to it. The parents, however, were anxious that something should be attempted, and after deliberating on the subject for some time, and considering the little success which had attended the treatment by puncturing and compressing, I concluded to try the *ligature.* Accordingly, when the child was about four months old, I placed loosely round the neck of the tumor a ligature of soft silk; and at the end of 24 hours, replaced it with another, which I drew a little tighter; directing the mother to cut it, if in my absence the child should seem threatened with convulsions. The next day, finding it had borne the ligature pretty well, I applied a second, and drew it tolerably tight. This, I found before leaving the house, was a source of considerable irritation, as the child became restless and fretful; but as it manifested no signs of cerebral disease, I did not relax the tension, and left it, simply directing its mother to keep it as quiet as possible, but not to give it an anodyne, as a medicine of that kind might give a tendency to compression of the brain. The following day I found my little patient quite tranquil, and learned that it had rested easy from the end of the first hour after I had left it.

I now determined to make the last application, and carrying my ligature as close as possible to the spine, drew it so tight as completely to strangulate the tumor. The child screamed, and was manifestly in great pain, but I left it with an injunction to the mother to not remove the ligature unless convulsions come on. It continued very fretful for several hours, but when I made my visit the succeeding day I found it in a quiet and comfortable sleep. On examination, I found the skin of a dark hue, indicating that the strangulation was perfect; and I simply directed a cooling aperient. On the succeeding day, I removed the whole tumor with the scalpel, cutting near to the ligature, and found that it contained about four ounces of yellowish serum. I suffered the ligature to remain, and covered the wound with slips of adhesive plaster, drawing the integuments as much as possible over the cut surface. These dressings were left on till pus began to appear. On removing them no orifice was perceptible, and in about 10 days, the ulcer cicatrized. Upwards of two years have elapsed since the operation, throughout which, the child has generally enjoyed good health, and is in all respects well developed.

*Cincinnati, 2d mo.*, 1337.

### Editorial Note.

We do not know that the ligature has not before been employed in the treatment of spina bifida, but its use in this case, by our townsman, Dr. Judkins, was doubtless with him, original; and the perfect success of the experiment should command the attention of the profession. It would manifestly not be adapted to tumors of a broad base. That in which he employed it, had a neck, and it was therefore practicable to bring the serous surfaces in contact. Being thus approximated, adhesion would take place, if *moderate* inflammation were excited, and this was accomplished by his ingeniously applying a ligature and lightened it gradually, so as to avoid that cutaneous irritation and inflammation, which might have required its removal, before internal adhesion had taken place.

